# Subcortical amygdala pathways enable rapid face processing

**DOI:** 10.1016/j.neuroimage.2014.07.047

**Published:** 2014-11-15

**Authors:** Mona M. Garvert, Karl J. Friston, Raymond J. Dolan, Marta I. Garrido

**Affiliations:** a Wellcome Trust Centre for Neuroimaging, University College London, 12 Queen Square, London WC1N 3BG, UK; b Queensland Brain Institute, The University of Queensland, St Lucia, 4072 Brisbane, Australia; c Australian Research Council Centre of Excellence for Integrative Brain Function, Australia

**Keywords:** Dynamic causal modelling, Subcortical processing, Amygdala, Connectivity, MEG

## Abstract

Human faces may signal relevant information and are therefore analysed rapidly and effectively by the brain. However, the precise mechanisms and pathways involved in rapid face processing are unclear. One view posits a role for a subcortical connection between early visual sensory regions and the amygdala, while an alternative account emphasises cortical mediation. To adjudicate between these functional architectures, we recorded magnetoencephalographic (MEG) evoked fields in human subjects to presentation of faces with varying emotional valence. Early brain activity was better explained by dynamic causal models containing a direct subcortical connection to the amygdala irrespective of emotional modulation. At longer latencies, models without a subcortical connection had comparable evidence. Hence, our results support the hypothesis that a subcortical pathway to the amygdala plays a role in rapid sensory processing of faces, in particular during early stimulus processing. This finding contributes to an understanding of the amygdala as a behavioural relevance detector.

## Highlights

• Face processing involves a functional subcortical route to amygdala.
• Subcortical visual pathway to amygdala is crucial during early visual processing.
• Visual processing of late time‐periods can also be explained by cortical connectivity.

## Introduction

Rapid detection of salient stimuli in the environment is of crucial importance for the survival of an organism. Humans can infer the valence of environmental cues directly, but they also infer valence from the reaction of others, in particular from their facial expressions. The amygdala contributes to the automatic detection of emotional, social or threatening stimuli (Anderson et al., 2003) or facial expressions (Santos et al., 2011) as well as the subsequent adaptation of behavioural responses (Haubensak et al., 2010). Little is known, however, about how relevant information reaches the amygdala so quickly. One model suggests that visual information about faces or whole bodies – particularly in a fearful or threatening context – is conveyed to the amygdala by a cortical and a subcortical processing route (De Gelder et al., 2004, Morris et al., 1998, Rudrauf et al., 2008, Vuilleumier et al., 2003). This is thought to enable rapid and automatic information processing more so than a resource-dependent cortical route (Tamietto and de Gelder, 2010). Diffusion tensor imaging has provided anatomical evidence for a subcortical visual pathway to the amygdala (Tamietto et al., 2012), and a subcortical route has been shown to be functionally active during auditory information processing (Garrido et al., 2012). However, the functional importance and mechanistic contribution of a subcortical connection has been questioned (Kumar et al., 2012, Pessoa, 2005). An alternative account suggests that a cortical route alone is sufficient, and the amygdala acts to allocate processing resources (Pessoa and Adolphs, 2010). Thus, the issue of whether a subcortical route to the amygdala is engaged during face processing remains unresolved.

Dynamic causal modelling (DCM) is a powerful approach for testing competing hypotheses about connectivity between brain areas. DCM is based on biologically plausible models of distributed and coupled neuronal dynamics on the millisecond timescale. By inverting these models one can estimate connection strengths and evaluate the evidence for different models of connectivity (Daunizeau et al., 2009, David et al., 2006, Friston et al., 2003). In this study, we used DCM to model magnetoencephalographic (MEG) event-related fields (ERFs) in response to emotional faces to test effective connectivity models of visual processing. Faces evoke a pronounced and well-characterised MEG component over the occipitotemporal cortex (Gao et al., 2012, Xu et al., 2005) and are known to elicit amygdala activity early after stimulus onset (Santos et al., 2011). We hypothesised that a subcortical connection subserves this early amygdala processing and therefore contributes to early ERF components, whereas a cortical model that precludes subcortical connections would only be sufficient to explain later components.

## Materials and methods

### Participants

Twelve neurologically healthy and naïve participants took part in the study (3 males, 9 females, age range 23–35 years). All participants reported normal or corrected-to-normal vision and normal hearing. The experimental procedure was approved by the University College London Hospitals Ethics Committee and written informed consent was obtained from all participants. Participants were remunerated for their time.

### Experimental procedure

Whole-head magnetoencephalography (MEG) data were recorded using a CTF 275-channel system with 274 functioning second-order axial gradiometers arranged in a helmet-shaped array. Signals were sampled at 600 Hz. To monitor head position with respect to the MEG sensors, three electrical coils were attached to the fiducials (nasion, and left and right preauriculars). Auditory stimuli were presented binaurally through headphones connected to piezoelectric transducers, which were positioned approximately 1 m below the sensor array. Participants were placed in front of a computer screen in a magnetically shielded room.

**Fig. 1:**
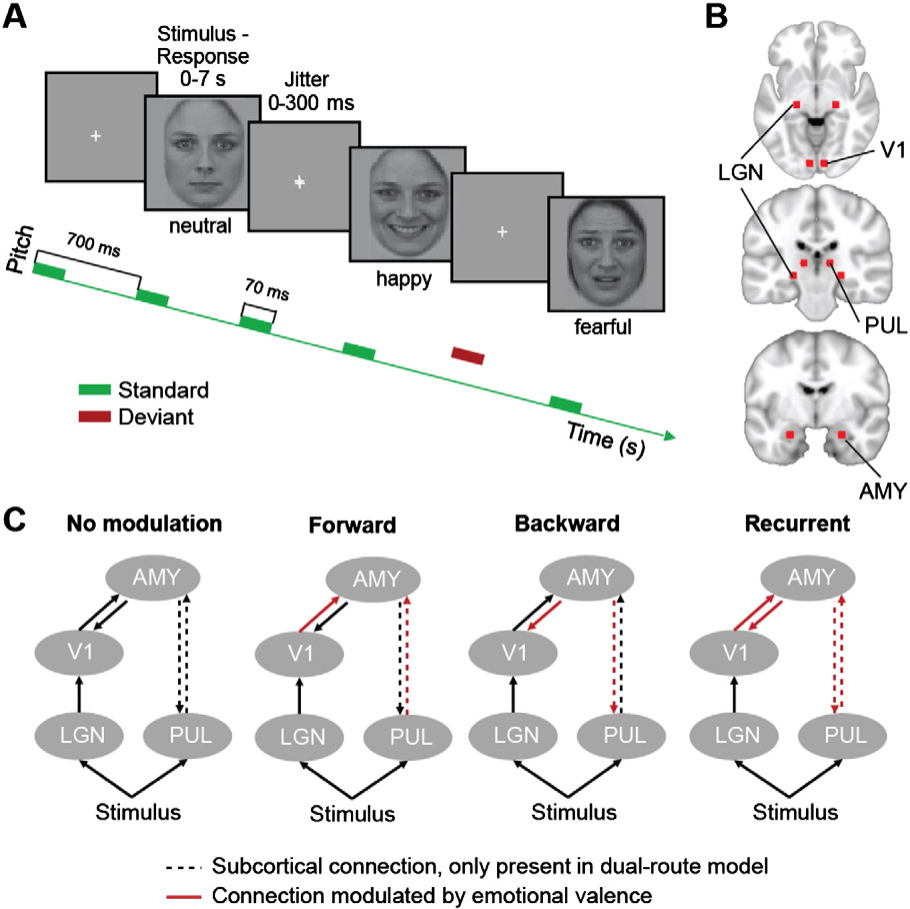
Behavioural task and DCM architecture. A Task structure. Participants performed a gender discrimination task on neutral, happy, or fearful faces presented in a randomised order. Concurrently, a task-irrelevant sequence of repetitive standard tones (green boxes) occasionally interrupted by deviant tones of higher pitch (red box) was presented binaurally. B Location of equivalent current dipoles included in the DCMs: lateral geniculate gyrus, LGN; primary visual cortex, V1; pulvinar, PUL and amygdala, AMY (See Materials and methods for coordinates). C Two model families were constructed: One family of models comprising a pulvinar–amygdala connection (dual-route models) and one family without this connection (cortical-only models). The dashed line indicates the presence/absence of this subcortical connection. We tested four patterns of modulation by emotional valence for both model families: (1) No modulation, (2) forward, (3) backward and (4) recurrent modulation between pulvinar/V1 and amygdala (indicated by red colouring of the arrows).

As reported in Garrido et al. (2012), participants performed a gender discrimination task on visually presented faces by button press (Fig. 1A). 27 male and 27 female faces with neutral, happy or fearful expressions were presented in random order with a total number of 99 faces per emotional condition. Faces were presented for 7 s each with an inter-trial interval jittered between 0 and 300 ms. In addition, tones were presented via headphones in 700 ms intervals for a period of 70 ms each. The tones were pure sinusoids of a particular standard frequency, sporadically interrupted by tones of a deviant frequency. Tones were not time locked to the onset of the visual stimuli and constituted an incidental oddball paradigm that has been reported elsewhere (Garrido et al., 2012). Participants were instructed to ignore the auditory stimulation and were repeatedly reminded to fixate the centre of the visual screen at all times. Stimuli were presented with the Cogent 2000 toolbox for MATLAB (http://www.vislab.ucl.ac.uk/cogent.php).

### Data pre-processing and analysis

Data were down-sampled to 200 Hz, band-pass filtered from 0.5 to 30 Hz and baseline corrected with reference to the interval −200 to 0 ms before the onset of visual stimuli. Subsequently, data were epoched with a time window of −200 to 600 ms with 0 ms denoting image onset. Signal contaminated by eye movements or muscular activity was removed using robust averaging as implemented in SPM8 (Wager et al., 2005). Trials were sorted according to emotional valence of the facial expression. Data preprocessing and analysis were performed with SPM8 (Litvak et al., 2011).

To test for differences in peak amplitude between the three emotional conditions, we first conducted paired t-tests over the whole sensor space and all time points to investigate whether fearful faces had a more pronounced ERF than happy or neutral faces. This requires stringent corrections for multiple comparisons. To increase statistical power, we also applied a sensor of interest approach as reported in Xu et al., 2005, which is comparable to a region of interest (ROI) analysis in fMRI. Sensors where the ERF evoked by faces was significantly greater than baseline (P < 0.05) for at least a time window of 25 ms centred on the peak response (and within the time window 0–300 ms) constituted our sensors of interest (SOI). This procedure prevents a bias in sensor selection in relation to the (orthogonal) effect of interest because it averages over neutral, happy, and fearful faces. Subsequently, a mean ERF was computed for the sensors of interest; the sign of the ERF recorded by sensors in the right hemisphere was reversed to match polarities. The relevant time period of interest was identified as the time window for which the mean ERF was significantly larger than baseline (p < 0.05). To test for condition-specific effects, the responses during this time period were averaged over all sensors of interest, to produce one value for each subject and condition. We expected average responses to be more pronounced for the fearful faces and performed two-sided paired t-tests to test this hypothesis.

### Dynamic causal modelling specification and Bayesian model selection

We used DCM to characterise changes in neuronal architecture underlying the electromagnetic signals observed in response to the presentation of faces. We tested two families of models (Penny et al., 2010) to address the contribution of a functional subcortical pathway to the amygdala: a family of dual-route models and a family of cortical-only models (Fig. 1C, according to Pessoa and Adolphs, 2010). Both families included eight sources, modelled as equivalent current dipoles (with no constraints on the symmetry of dipolar orientation). The prior locations of sources were taken from the literature (Fig. 1B). They comprised the lateral geniculate nuclei (LGN, MNI coordinates left: − 22, − 22, − 6; right: 22, − 22, − 6 according to Kastner et al., 2004; similar coordinates reported in Hess et al., 2009, Mullen et al., 2010, Schneider et al., 2004), the primary visual cortex (V1, left: − 7, − 85, − 7; right: 7, − 85, − 7 according to Hasnain et al., 1998, Wohlschläger et al., 2005), the pulvinar (PUL, left: − 12, − 25, 7; right: 12, − 25, 7 according to Kastner et al., 2004; similar coordinates reported in Beer et al., 2002 and Villeneuve et al., 2005), and the amygdala (AMY, left: − 23, − 5, − 22; right: 23, − 5, − 22 according to Canli et al., 2002, similar coordinates reported in Morris et al., 1998, Vuilleumier et al., 2003, Williams et al., 2001). Input sources for visual information were left and right lateral geniculate nucleus and pulvinar. In the cortical-only models, visual information reached the amygdala only— via the lateral geniculate nucleus and the primary visual cortex. In the dual-route models, the amygdala received additional input from the pulvinar, i.e. a functional cortical and a subcortical pathway operated in parallel (Fig. 1C).

Models were compared statistically across participants using random-effects Bayesian model selection (BMS, Stephan et al., 2010). To investigate whether the subcortical pathway was recruited at particular points in time we repeated this analysis as a function of the post-stimulus time window (Rudrauf et al., 2008): We assessed the time specific contribution of the subcortical pathway by comparing the evidence for models with and without a subcortical route while increasing the analysis time window from 0–60 ms in 10 ms steps up to 0–300 ms (Auksztulewicz et al., 2012, Garrido et al., 2007).

The ERF evoked by fearful faces was found to have slightly higher amplitude than the ERF evoked by happy, but not neutral faces. This suggested that we should be most sensitive to emotion-specific differences in connectivity by comparing happy vs. fearful faces. Therefore, we contrasted evoked responses caused by the presentation of happy vs. fearful faces to assess valence-specific differences in connectivity. We compared evidence for both the dual-route and the cortical-only models with: (1) no valence-specific modulation, (2) valence-specific modulation of forward projections to the amygdala, (3) of backward projections from the amygdala, and (4) of recurrent connections (Fig. 1C).

### DCM contribution analysis

We performed a contribution analysis to evaluate the impact of the forward connection from the pulvinar to the amygdala on both pulvinar and amygdala sources under the dual-route models. This contribution analysis provides a simulation for the time courses of amygdala and pulvinar source activity that we would see were we able to enforce a small increase, 0.0025, in the pulvinar-to-amygdala connection. This analysis was performed over the post-stimulus time window of 300 ms. The predicted activity at the source level was subsequently averaged over models and participants to quantify the effect at the population level.

## Results

**Fig. 2:**
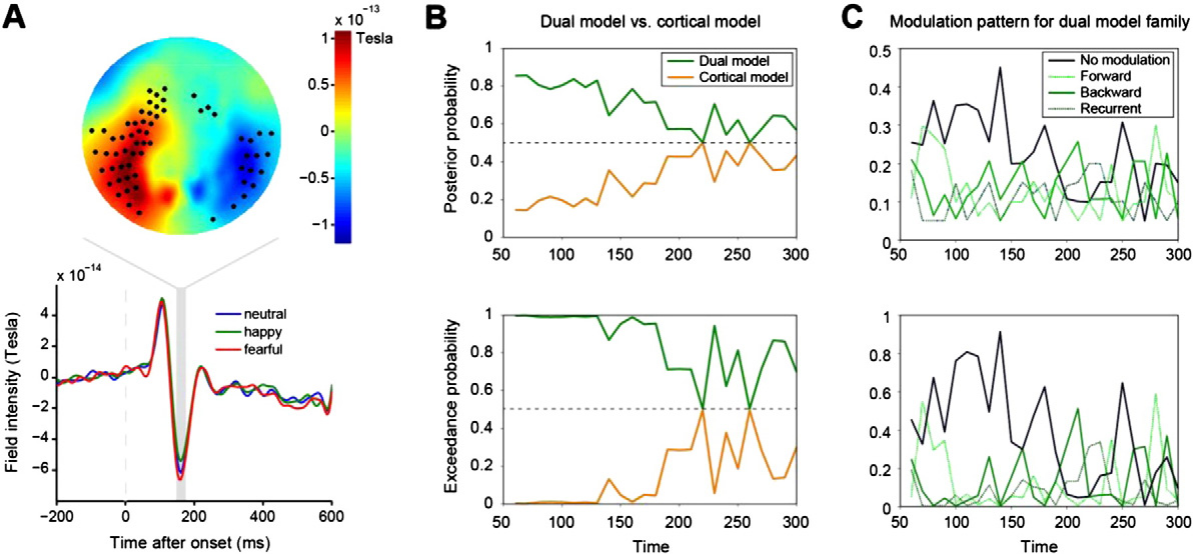
Face-evoked field and DCM results. A Spatial distribution of evoked fields elicited by the presentation of faces reveals a clear M170 component located over the occipito-temporal cortex (top). Sensors of interest (SOIs) with a significant face-evoked component are indicated by dots (see Materials and methods for details). When averaged over participants and all SOIs, the component was slightly larger for fearful than happy faces (bottom). B Posterior and exceedance probability for the dual-route and the cortical-only models pooled over modulation patterns over post-stimulus time intervals. The dual-route model family explains the data better for shorter data segments (< 180 ms), whereas model evidence is equal for both model families for longer data segments (> 180 ms). C Posterior and exceedance probabilities for dual-route models with no/forward/backward or recurrent modulation of connectivity by emotional context. We found no clear evidence for emotion-specific modulation.

To examine whether emotional faces reach the amygdala via a dual-route (subcortical and cortical pathway) or via a cortical pathway only, we recorded MEG data from 12 subjects, while they performed a gender discrimination task for neutral, happy and fearful faces. We did not find differences in response times across emotional valence (neutral: 1004 ± 90 ms, happy: 1001 ± 89 ms, fearful: 991 ± 88 ms; mean ± s.e.m., repeated measures ANOVA, F(2,22) = 0.38, p = 0.7). The grand-average ERF response showed a typical face-evoked potential peaking at around 170 ms after stimulus onset — the M170 component (Fig. 2A; Xu et al., 2005, Gao et al., 2012).

We hypothesised that the M170 component would be more pronounced for fearful faces than for neutral and happy faces. However, a paired *t*-test for differences between the fearful and the two other conditions over sensor-space and all post-stimulus times failed to reach significance after correction for multiple comparisons over all sensors and time points. A sensor of interest analysis (Xu et al., 2005) identified 62 out of 274 channels, mostly located over the occipitotemporal region, that exhibited a significant response to faces (p < 0.05, Fig. 2A). To finesse the multiple comparison problem, we computed the mean ERF over these face sensitive sensors for each subject and condition for the time window 150–190 ms. Two-sided paired t-tests of these subject specific response averages were significant when comparing fearful and happy (t(11) = 1.98, p = 0.036), but not when comparing fearful and neutral (t(11) = 0.63, p = 0.27). The evidence for differential processing of emotional valence in the context of our particular paradigm is therefore weak.

Previous studies have suggested that information about emotional faces reaches the amygdala very rapidly, as an amygdala response can be detected around 140 ms after face onset (Cornwell et al., 2008). To test for a subcortical pathway between the pulvinar and the amygdala involved in this fast information transmission, we used dynamic causal modelling. We compared the model evidence for a dual-route model family, comprising a cortical as well as a subcortical connection and a cortical-only model family comprising a cortical pathway alone. Our models included four sources known to be involved in face processing: the lateral geniculate nucleus (LGN), the visual cortex (V1), the pulvinar (PUL), and the amygdala (AMY, Fig. 1B).

Our models include some deep subcortical structures located where MEG sensitivity drops relative to more superficial cortical regions. To assess the sensitivity of our MEG system to these structures, we performed simulations. According to our simulations, MEG sensitivities to LGN, pulvinar, and amygdala, relative to the primary visual cortex, were 0.78 ± 0.07, 0.46 ± 0.04, and 0.86 ± 0.08, respectively (see Supplementary Material for details). This demonstrates sensitivities in the amygdala and LGN were comparable with V1, but sensitivity was lower in the pulvinar. Importantly, these differences in sensitivity do not affect DCM. DCM accommodates different sensitivities to different sources, because this differential sensitivity is an integral part of the model (which include the electromagnetic lead fields). As a consequence, DCMs with hidden sources (with no sensitivity) can yield better models than models without (David et al., 2011).

In both model families, LGN, V1, pulvinar and amygdala were present in both hemispheres (Fig. 1B) and the respective structures in the left and right hemispheres were not connected. The circuitry received inputs via the pulvinar and the LGN, and the LGN projected to the amygdala via V1 (Fig. 1C). The dual-route model family contained an additional direct connection from the pulvinar to the amygdala. We hypothesised that the small difference in ERFs for fearful vs. happy faces might be related to emotion-specific modulation of the connectivity between the areas included in our models. Therefore, we modelled condition-specific differences in connectivity for fearful vs. happy faces by defining four models within both the dual-route and the cortical-only model families: a model with (1) no modulations, (2) modulation of forward connections, (3) modulation of backward connections or (4) modulation of forward and backward connections by emotional valence. Neutral faces were not included in this analysis because there was no evidence for differential processing of neutral vs. happy or neutral vs. fearful faces in the ERFs.

In a first analysis, we pooled over modulation patterns and applied random-effects Bayesian model selection (BMS) to compare evidence for dual-route and cortical-only models as a function of the post-stimulus time interval. Time intervals ranged from 0–60 ms to 0–300 ms. The dual-route model family had higher posterior and exceedance probabilities for early intervals of up to ca. 180 ms window length (Fig. 2B). For longer intervals, the cortical-only model family had a roughly equivalent exceedance probability. This indicates that the subcortical connection plays a crucial role in early, but not necessarily in late stimulus processing. It is unlikely that this effect is biased by the particular model architecture we chose, because the results were similar when assessing the model evidence for alternative model architectures comprising interhemispheric connectivity, unilateral midline structures or intrinsic amygdala connectivity: early time periods were best explained by the dual-route model family when pooled over all model architectures, whereas model evidence at later time periods was comparably high for the cortical-only model family (see Supplementary Text T2 and Fig. S2 for details). Having said this, the original model architecture (bilateral midline structures, no interhemispheric connectivity, no intrinsic amygdala connectivity) had the highest model evidence of the 12 model architectures we assessed.

To test whether emotional valence differentially recruits the circuitry, we compared model evidence for the four family members of the dual-route model family (original model architecture) over time. The models comprised a model with (1) no modulation, (2) forward, (3) backward, and (4) recurrent modulation by valence (Fig. 1C). At early time points, we found slightly higher posterior and exceedance probabilities for the model without any modulation than for the models with modulation (Fig. 2C). At no time point could we find convincing evidence for emotion-specific modulation; the posterior and exceedance probabilities were comparable for all models. We performed the same analysis for the cortical-only model family and found no evidence for emotion-specific modulation either (not shown for simplicity). This suggests that the subcortical connection is important for processing faces in general, but is not recruited specifically for processing negative emotional valence.

**Fig. 3:**
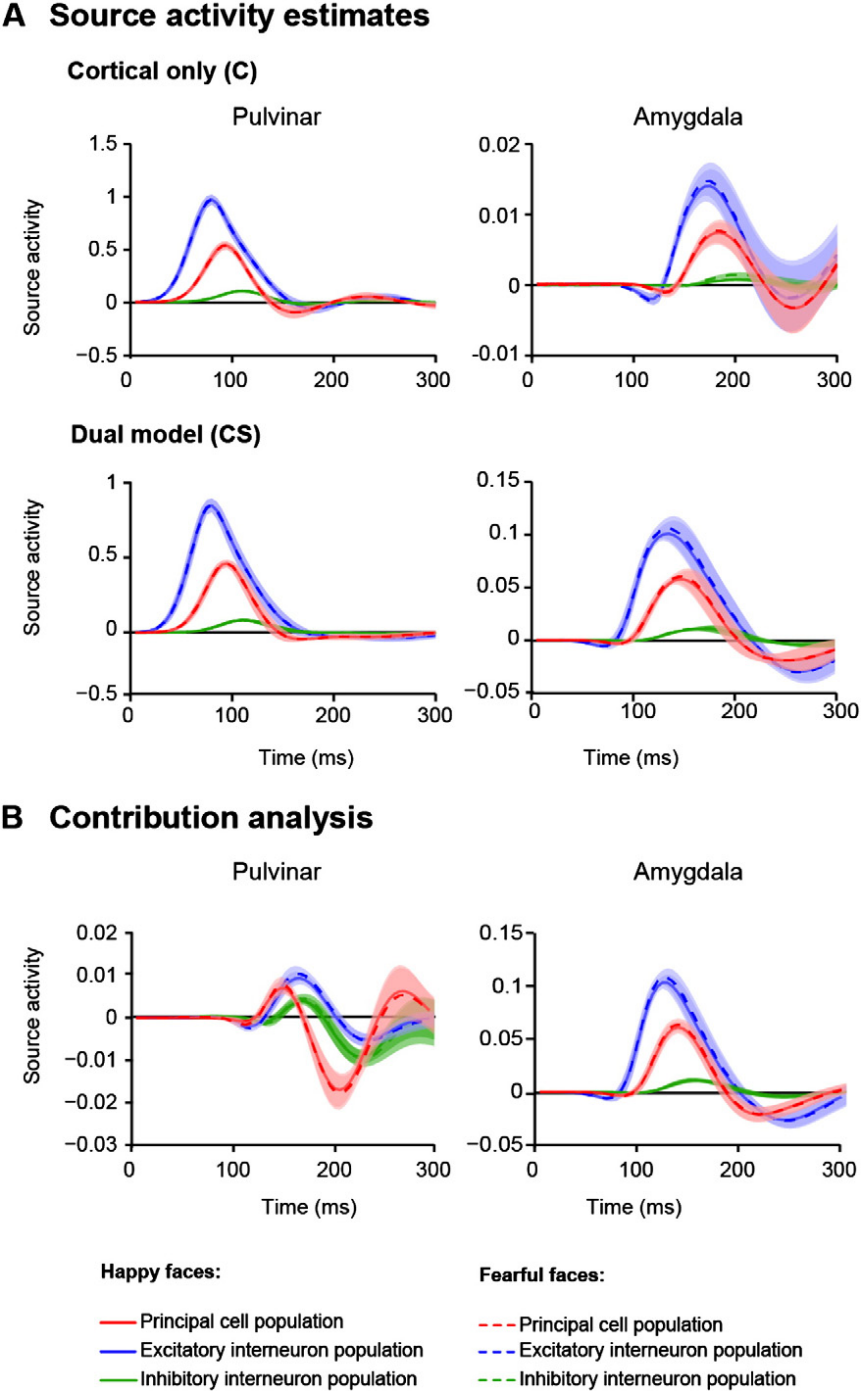
DCM source activity estimates and contribution analysis. A DCM source activity estimation for happy and fearful faces for the dual-route and cortical-only model families (mean/s.e.m.). Each microcircuit is modelled by a population of excitatory principal cells which receives input from inhibitory and excitatory interneuron populations (Jansen and Rit, 1995). Activity is averaged over modulation pattern and participants. B Contribution analysis. Changes in activity in the pulvinar and amygdala in response to an incremental change in pulvinar–amygdala connectivity (mean/s.e.m.) are shown. Only the left hemisphere is depicted in A and B, as the results for the right hemisphere were very similar. Solid line: response to happy faces, dashed line: response to fearful faces, red: activity of principal neuron population, blue: activity of excitatory interneuron population, green: activity of inhibitory interneuron population.

We hypothesised that the general difference in model fit for the dual-route vs. cortical-only model families should become apparent as a difference in estimated amygdala source activity. To examine this issue quantitatively, we looked at the source activity for the two model families as predicted by the DCMs (Fig. 3A). Activity in V1 and LGN did not differ for the dual-route and cortical-only model families. Furthermore, we observed comparable activity in the pulvinar for both types of model families. The principal cell population reached its peak activity at around 100 ms, then declined to negative values at around 150 ms and then slowly returned to baseline. Source activity in the amygdala, however, peaked at around 140 ms in the dual-route model, but substantially later, at 180 ms, in the cortical-only model (Fig. 3A). In addition, the amplitude of amygdala activity in the dual-route model was increased tenfold in comparison to the cortical-only model. This underlines the importance of a subcortical input to the amygdala in face processing.

The dual-route account predicts that perturbations to the connection between the pulvinar and the amygdala should substantially alter the estimated amygdala response. To assess this prediction, we simulated the changes in pulvinar and amygdala source activity caused by small increases in the connectivity between the pulvinar and the amygdala for the dual-route models (Fig. 3B). With this analysis we were able to quantify the effect that network connectivity, more specifically the forward connection pulvinar-to-amygdala, has on pulvinar and amygdala source activity. Amygdala activity was altered dramatically under this perturbation, illustrating the role of the subcortical route in engaging and enhancing amygdala activity. In addition, alterations were also found at the level of the pulvinar. The peak contribution effect in the pulvinar was expressed positively at 145 ms and negatively at around 200 ms. Crucially, the peak for the amygdala occurred at around 135 ms; i.e., even though the amygdala receives forward afferents from the pulvinar and is thus located at a higher level in the hierarchy the peak occurred earlier than in the pulvinar (Fig. 3B). This reflects the fact that the contribution of forward connectivity to pulvinar responses is mediated by recurrent backward connections from the amygdala.

In line with the lack of evidence for emotion-specific modulation of the circuitry, we also found that the source activity and the results of the contribution analysis were indistinguishable for fearful and happy faces. This suggest that the subcortical connection is of general importance for processing faces and it is not specifically recruited for processing fearful information (Garrido et al., 2012, Pegna et al., 2005).

## Discussion

We used dynamic causal modelling (DCM) to investigate the functional role of a putative subcortical pathway to the amygdala in processing emotional faces. We found that a subcortical connection between the pulvinar and the amygdala plays a role in early, but not late, visual processing.

It is known that the amygdala reacts rapidly to salient stimuli, such as faces with an emotional expression, even when these are presented for a period too short for conscious perception (Jiang et al., 2009, Morris et al., 1998, Whalen et al., 1998). Furthermore, patients with lesions to their visual cortex can correctly guess the emotional expression, but no other characteristics, of faces (Pegna et al., 2005). Based on these observations, and on the presence of an anatomical connection from subcortical areas to the amygdala in rodents (Campeau and Davis, 1995, Shi and Davis, 2001) and humans (Tamietto et al., 2012), a subcortical route to the amygdala has been proposed to enable fast and efficient information processing (De Gelder et al., 2004, Tamietto and de Gelder, 2010). In this view, connections of the amygdala to a wide range of cortical areas, ranging from the thalamus to the orbitofrontal cortex, the anterior insula and the anterior cingulate cortex, allow for rapid adaptive behavioural responses to the detection of a salient stimulus.

However, this notion has been challenged by the observation that the amygdala was not essential for rapid fear detection in one individual with bilateral amygdala lesions (Tsuchiya et al., 2009). Furthermore, subcortical processing of emotional stimuli is not necessarily faster than cortical processing in monkeys suggesting that short-latency responses observable in the amygdala might not originate from a subcortical pathway (Gothard et al., 2007, Lamme and Roelfsema, 2000). Furthermore, electrophysiological recordings in humans have shown that response latencies of amygdala neurons are no faster than entorhinal and hippocampal latencies, which are presumably generated along cortical connections (Mormann et al., 2008). Based on these findings, a ‘multiple waves’ model has been proposed as an alternative to the standard ‘dual-route’ hypothesis (Pessoa and Adolphs, 2010). According to the ‘multiple waves’ model, cortical routes bypass various cortical areas that are assumed to be critically involved in visual processing and thereby ensure fast information transmission, without a subcortical connection. This model assumes that the amygdala receives pre-processed information, and its role is relegated to mere modulation of stimulus processing in visual areas and allocation of resources to ensure efficient processing of relevant information.

We found that stimulus processing during early time periods is best explained by a neuronal architecture containing a subcortical connection from the pulvinar to the amygdala, independent of the stimulus' emotional valence. In later stimulus processing, the evidence for a model without this subcortical connection was similar to the evidence for the dual-route model. Comparable time-dependent contributions of a subcortical pathway to information processing have been demonstrated in the auditory system (Garrido et al., 2012). Based on these findings, we conclude that a subcortical connection to the amygdala plays a functional role in early face processing. This is in line with a number of anatomical (Tamietto et al., 2012) and functional imaging studies (De Gelder et al., 2004, Liddell et al., 2005, Morris et al., 1998, Vuilleumier et al., 2003). A two-pathway architecture has also been shown to provide an accurate description of emotional visual processing in a previous DCM study (Rudrauf et al., 2008). Our results extend these findings by demonstrating a time-dependent contribution of the subcortical connection between the pulvinar and the amygdala to visual processing.

Processing speed in the amygdala in response to sensory input is still under debate. Whereas some electrophysiological studies in humans have found amygdala peak activity as early as 40 ms after stimulus onset (Luo et al., 2010) and very fast face processing (Meeren et al., 2013), other studies report long latency responses in the amygdala with a time course more similar to activation via a cortical pathway (Gothard et al., 2007, Luo et al., 2010, Mormann et al., 2008). However, without an explicit network model or causal manipulation it is difficult to know whether the observed amygdala peak activity results from forward or recurrent activity. If a response peak in the amygdala is due to recurrent activity, it would likely have a similar latency to a cortical response peak. Indeed, we observed a second (negative) peak in the amygdala that is likely to be caused by recurrent activity. In general, our modelling results suggest that amygdala activity under the cortical-only model is weaker and arises at a later time point than under the dual-route model.

We observed emotional modulation only for the comparison of peak amplitude for fearful and happy faces. The difference in the MEG signal did not survive multiple comparison correction, we did not find an effect in the underlying connectivity, and the contribution analysis did not reveal differential modulation for neutral and fearful stimuli. This finding is in line with the notion of the amygdala as a ‘behavioural relevance detector’ which processes salient stimuli, such as faces, irrespective of their emotional valence (Attar et al., 2010, Santos et al., 2011). The stimuli we used might be processed equivalently by the specific circuitry we analysed in this study, and emotion processing might differ only after emotional valence has been attributed by the amygdala by higher cognitive areas. It is important to note, however, that in our experiment we did not manipulate salience in a way that would allow inferences about processing of salient stimuli in general.

It is also conceivable that we do not find any difference for emotional valence in our DCM because of the incidental nature of the emotional valence in our task, where information about the emotional valence was not relevant for discriminating gender. A task that requires explicit recognition of an emotional attribute and thereby directs attention to the emotion might cause top–down modulations of circuit dynamics, although presumably at later processing stages (de Gelder et al., 2012). Similarly, top–down inhibition of amygdala activity can be observed under high task load (De Gelder et al., 2012, Mitchell et al., 2007, Silvert et al., 2007). Nevertheless, it is unlikely that the incidental nature of our emotion manipulation explains a failure to observe differential effects. Indeed, emotion is processed when it is irrelevant for task performance (Vuilleumier et al., 2001) and when subjects are not aware of the nature of the stimulus (Liddell et al., 2005, Morris et al., 1998, Whalen et al., 1998).

An alternative explanation for the lack of differentiation between emotional states is an insufficient sensitivity of our experimental design for assessing the role of emotional valence in recruiting a subcortical pathway. This assumption is corroborated by the observation that we do not find amygdala-induced modulation of source activity in the visual cortex. Our task was optimised for assessing the contribution of a subcortical connection to face processing in general, and it is possible that a greater number of trials per emotion would have revealed an effect of valence. To draw conclusions about the emotional modulation of connections with higher sensitivity, a more efficient experimental design is required.

It may seem surprising that we use MEG to make claims about subcortical sources that are so close together. However, a growing number of empirical papers highlight source activity detected using MEG originating from disparate deep structures such as the amygdala, the hippocampus and even the pulvinar and the thalamus (Attal et al., 2009, Attal et al., 2010, Cornwell et al., 2008, David et al., 2011, Dumas et al., 2010, Dumas et al., 2011, Dumas et al., 2013, Garrido et al., 2012, Guitart-Masip et al., 2013, Luo et al., 2007, Moses et al., 2007, Parkkonen et al., 2009, Poch et al., 2011, Quraan et al., 2011, Tesche and Karhu, 2000a, Tesche and Karhu, 2000b). A recent study even localised differential effects of valence and arousal in amygdala subdivisions (Styliadis et al., 2013). These empirical findings are corroborated by a theoretical study demonstrating in anatomically realistic simulations that activity in deep brain regions such as the hippocampus and the amygdala can be recorded using MEG (Attal and Schwartz, 2013). This is rendered feasible by higher current densities generated within these deep areas compared to what is the case for the neocortex, which compensates for a greater distance to the sensors. Localisation errors in the hippocampus lie between 0.5 and 2 cm in simulations, which suggests that a spatial resolution in deep sources is typically less than 2 cm. Distances between the structures considered in our models are mostly larger than 2 cm, with the exceptions of the two V1 (1.4 cm), as well as between LGN and pulvinar (1.67 cm). Nevertheless, our model comparisons show that bilateral V1 explains the data better than a single V1.

The astute reader will notice that we have used a classic neuron mass model for subcortical sources in our DCM (Jansen and Rit, 1995). This model is based upon a cortical canonical microcircuit, which – at first glance – may not appear to be appropriate for subcortical structures. However, there is a remarkable consistency in terms of the composition of neuronal populations and their intrinsic connectivity between many cortical and subcortical microcircuits. For example, the lateral geniculate contains three populations (magnocellular, parvocellular and koniocellular cells) that we associate with the three populations of our neural mass model. When modelling subcortical sources, we assume that the pyramidal population models the principal cells in each subcortical source of extrinsic afferents. Clearly, one could consider bespoke neuron mass models of subcortical structures (of the sort considered by Moran et al. (2011) for the cortico-basal ganglia–thalamocortical loop); however, we considered the standard (three population) model sufficient for our particular question — given the usual caveats about inferences using DCM (namely, that these inferences are only as good as the models considered).

With regard to LGN–pulvinar we acknowledge that there is a possibility that we are picking up activity from a nearby region. This is mitigated by the fact that the spatial resolution of MEG is much less of an issue for DCM than for source reconstruction, where strong a priori hypotheses can be drawn from a body of extant research. Spatial resolution does not affect the inversion of electromagnetic forward models implicit in DCM, because source locations are predefined and DCM does not need to detect a source directly. A deep source that has effects on evoked responses in (detectable) superficial sources will still contribute to the data, such that the contribution of deep sources to evoked responses can be assessed. This is a fundamental advantage of DCM over classic electromagnetic reconstruction procedures that do not have a forward model of coupling among sources. In short, the validity of DCM is not compromised by the ability to record high signal-to-noise ratio data from all sources present in the model (Attal et al., 2012). Indeed, others have successfully used DCM to emulate hidden or silent sources (i.e., sources that do not contribute to the activity recorded at the scalp) in application to language models (David et al., 2011). Models with a hidden (thalamic) source explained the data much better than models without. However, we acknowledge that our anatomical labelling and interpretation of sources cannot be validated empirically using MEG. In other words, our hypotheses include the functional architectures (and anatomical designations) implicit in our forward model, and the ensuing model comparisons are therefore tests of these hypotheses.

In summary, our data demonstrate the importance of a functional visual subcortical pathway to the amygdala during early time periods of face processing, and thereby contribute to our understanding of the brain circuitry involved in salient information processing.
