# Supplementary material for: Subcortical amygdala pathways enable rapid face processing
Source: Neuroimage. 2014 Nov 15;102:309–16. doi: 10.1016/j.neuroimage.2014.07.047 (PMC4229499; doi:10.1016/j.neuroimage.2014.07.047)
Supplement: Supplementary file 1 — Supplementary material. [file mmc1.pdf]

Supplementary Material

Subcortical amygdala pathways enable rapid visual information processing

Mona M. Garvert, Karl J. Friston, Raymond J. Dolan and Marta I. Garrido

Inventory:

- Supplemental Text and Figure S1: Sensitivity to deep brain structures
- Supplemental Text and Figure S2: Dual-route vs. cortical model for other model architectures

Supplemental Text T1

Sensitivity to deep brain structures

Our DCMs include deep brain structures. To test whether sensitivity to signal originating in these structures was high enough in our group of participants, we compared the lead field magnitudes for dipoles within the LGN, the pulvinar, and the amygdala relative to V1, similar to prior work by Garrido et al. (2012). These simulations are based on the geometry of the cortical mesh for a single sphere head model.

According to our simulations, the sensitivities to LGN, pulvinar, and amygdala relative to the primary visual cortex were 0.78 ± 0.07, 0.46 ± 0.04, and 0.86 ± 0.08, respectively. This demonstrates that we do not lose much sensitivity in the amygdala and in the LGN when compared to V1, but sensitivity drops in the pulvinar (see Figure S1). This is consistent with findings by Attal and Schwartz (2013), who used simulations based on anatomically realistic models to show that activity in deep regions such as hippocampus and amygdala can theoretically be recorded with MEG. Current densities in these deep areas are higher than in the neocortex, which compensates the longer distance to the sensors. Importantly, localization errors in the hippocampus lie between 0.5 and 2 cm (Attal and Schwartz, 2013), suggesting that spatial resolution in deep sources is typically less than 2 cm. This is in line with a considerable number of papers demonstrating reliable reconstruction of deep source activity with MEG such as amygdala, hippocampus, thalamus, and pulvinar (Attal et al., 2010, 2009; Cornwell et al., 2008; David et al., 2011; Dumas et al., 2013, 2011, 2010; Garrido et al., 2012; Guitart-Masip et al., 2013; Luo et al., 2007; Moses et al., 2007; Parkkonen et al., 2009; Poch et al., 2011; Quraan et al., 2011; Tesche and Karhu, 2000a, 2000b). A recent study was even able to localise differential effects of valence and arousal in amygdala subdivisions (Styliadis et al., 2013).

Importantly, however, DCM does not rely on detectable sources only. Others have successfully used hidden sources with DCM to emulate silent sources, i.e. sources that do not contribute to the activity recorded at the scalp and yet contribute to model fit. Indeed it has been shown that models with a hidden subcortical source explained the data much better than models without it (David et al., 2011). The reason is that deep sources, which cannot be detected on the scalp level, will still contribute to the data if they affect activity in detectable superficial sources. The existence of a forward model of coupling among sources is an important advantage of DCM over other electromagnetic reconstruction procedures, because its validity is not comprised by the ability to record data from all sources present in the model (Attal et al., 2012).

Supplemental Figure S1

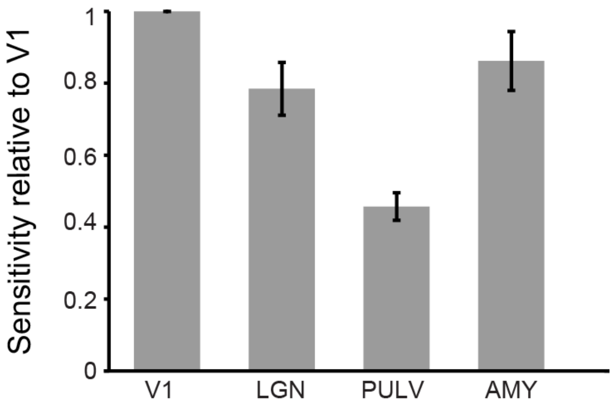

Figure S1. Sensitivity to signal from structures in our models relative to sensitivity to signal originating from V1.

Supplemental Text T2

Dual-route vs. cortical model for other model architectures

It is conceivable that our findings were biased by the particular model architecture we chose here (bilateral midline structures, no interhemispheric connectivity, no intrinsic connectivity in the amygdala). To examine whether our results are robust with respect to other model architectures with comparable prior probabilities, we constructed alternative model families. All models included equivalent current dipoles placed at coordinates corresponding to left and right LGN, V1, pulvinar and amygdala (see Methods section of main text for details). Model families differed in the following aspects:

- Families with bilateral or unilateral midline connectivity: In bilateral models, structures were connected as depicted in Figure 1C in both hemispheres. It is conceivable, however, that the sensitivity of the MEG system is not sufficient to differentiate between signals from equivalent structures in the right and the left hemisphere which are located close to the midline. Thus, in unilateral models, midline structures (pulvinar and V1) in one hemisphere were connected to bilateral amygdala and LGN and connectivity from the same structures in the other hemisphere was removed. For the left unilateral model this means that the left pulvinar was connected to both left and right amygdala, and left V1 was connected to left and right amygdala and LGN. The right pulvinar and V1 existed, but were not connected to any structure. The opposite applied to the right unilateral model.
- Families with or without interhemispheric connectivity: To test whether interhemispheric connectivity between structures in both hemispheres contributed to the signal, we tested model families with and without interhemispheric connections between the left and right LGN, V1, pulvinar and amygdala.
- Families with or without intrinsic connectivity in the amygdala: It is conceivable that intrinsic activity in the amygdala is of particular importance for processing salient

information because it enhances amygdala activity. Therefore, we also designed models with and without intrinsic amygdala connectivity.

We performed random-effects Bayesian model comparison to compare model evidence for the thus constructed twelve model families at the post-stimulus time [0 – 300] ms (pooled over dual-route vs. cortical models and modulation patterns). We found that the model that we had chosen for our main analysis (bilateral midline structures, no interhemispheric connectivity and no intrinsic amygdala connectivity) had the highest exceedance probability (Supplementary Figure S2A). However, to ensure that our inference regarding the importance of a subcortical connection between the pulvinar and the amygdala was not biased by the particular model architecture we chose, we compared model evidence for the dual-route and the cortical model families pooled over all 12 model families and modulation patterns. Importantly, we found a similar temporal evolution: Whereas posterior and exceedance probabilities were high for the dual model family at shorter post-stimulus times, at longer post-stimulus times both model families had comparable evidence (Figure S2B).

Supplemental Figure S2

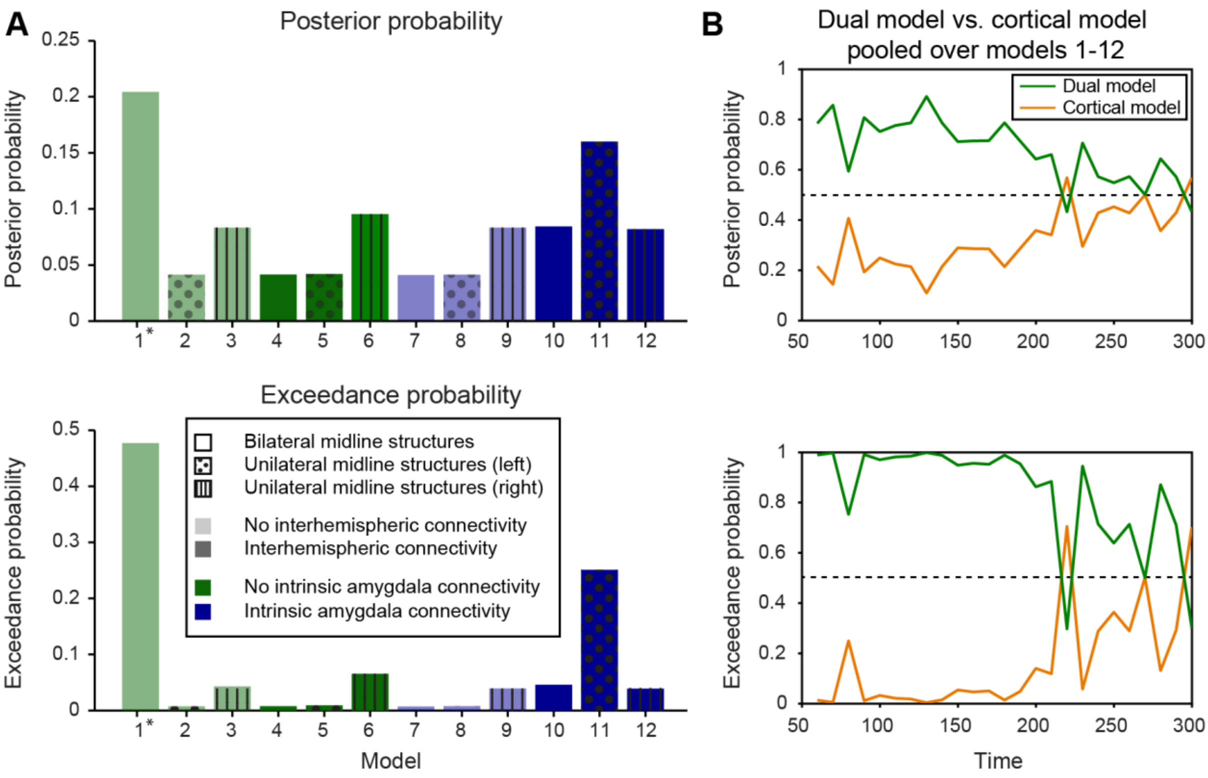

*Model architecture used in main analysis

Figure S2 (related to Figure 2). Model families with different model architectures. A Posterior and exceedance probability for different model architectures at a post-stimulus time interval of [0 – 300] ms. Model families were constructed according to Figure 1C, but varied in three aspects: bilateral/unilateral midline structures, interhemispheric connectivity and intrinsic amygdala connectivity. Results are pooled over dual-route and cortical-only models and modulation patterns. The model we used for the main analyses is model 1: bilateral midline structures, no interhemispheric connectivity, no intrinsic amygdala connectivity. **B** Posterior and exceedance probability separated for the dual-route and the cortical-only model family over post-stimulus time intervals. Results were pooled over models 1-12 and modulation patterns. In accordance with the results for model 1 only, the family of dual-route models explains the data better for shorter data segments whereas model evidence is comparable for both model families for longer data segments.

Supplemental References

Attal, Y., Bhattacharjee, M., Yelnik, J., Cottereau, B., Lefèvre, J., Okada, Y., Bardinet, E., Chupin, M., Baillet, S., 2009. Modelling and detecting deep brain activity with MEG and EEG. IRBM 30, 133–138.

Attal, Y., Maess, B., Friederici, A., David, O., 2012. Head models and dynamic causal modeling of subcortical activity using magnetoencephalographic/electroencephalographic data. Reviews in the Neurosciences 23.

Attal, Y., Schwartz, D., 2013. Assessment of subcortical source localization using deep brain activity imaging model with minimum norm operators: A MEG study. PLoS ONE 8, e59856.

Attal, Y., Yelnik, J., Bardinet, E., Chupin, M., Baillet, S., 2010. MEG detects alpha-power modulations in pulvinar, in: Supek, S., Sušac, A. (Eds.), 17th International Conference on Biomagnetism Advances in Biomagnetism – Biomag2010, IFMBE Proceedings. Springer Berlin Heidelberg, pp. 211–214.

Cornwell, B.R., Carver, F.W., Coppola, R., Johnson, L., Alvarez, R., Grillon, C., 2008. Evoked amygdala responses to negative faces revealed by adaptive MEG beamformers. Brain Res 1244, 103–112.

David, O., Maess, B., Eckstein, K., Friederici, A.D., 2011. Dynamic causal modeling of subcortical connectivity of language. J. Neurosci. 31, 2712–2717.

Dumas, T., Attal, Y., Chupin, M., Jouvent, R., Dubal, S., George, N., 2010. MEG study of amygdala responses during the perception of emotional faces and gaze, in: Supek, S., Sušac, A. (Eds.), 17th International Conference on Biomagnetism Advances in Biomagnetism – Biomag2010, IFMBE Proceedings. Springer Berlin Heidelberg, pp. 330–333.

Dumas, T., Attal, Y., Dubal, S., Jouvent, R., George, N., 2011. Detection of activity from the amygdala with magnetoencephalography. IRBM 32, 42–47.

Dumas, T., Dubal, S., Attal, Y., Chupin, M., Jouvent, R., Morel, S., George, N., 2013. MEG evidence for dynamic amygdala modulations by gaze and facial emotions. PLoS ONE 8, e74145.

Garrido, M.I., Barnes, G.R., Sahani, M., Dolan, R.J., 2012. Functional evidence for a dual route to amygdala. Curr. Biol. 22, 129–134.

Guitart-Masip, M., Barnes, G.R., Horner, A., Bauer, M., Dolan, R.J., Duzel, E., 2013. Synchronization of medial temporal lobe and prefrontal rhythms in human decision making. J. Neurosci. 33, 442–451.

Luo, Q., Holroyd, T., Jones, M., Hendler, T., Blair, J., 2007. Neural dynamics for facial threat processing as revealed by gamma band synchronization using MEG. Neuroimage 34, 839–847.

Moses, S.N., Houck, J.M., Martin, T., Hanlon, F.M., Ryan, J.D., Thoma, R.J., Weisend, M.P., Jackson, E.M., Pekkonen, E., Tesche, C.D., 2007. Dynamic neural activity recorded from human amygdala during fear conditioning using magnetoencephalography. Brain Res. Bull. 71, 452–460.

Parkkonen, L., Fujiki, N., Mäkelä, J.P., 2009. Sources of auditory brainstem responses revisited: contribution by magnetoencephalography. Hum Brain Mapp 30, 1772–1782.

Poch, C., Fuentemilla, L., Barnes, G.R., Düzel, E., 2011. Hippocampal theta-phase modulation of replay correlates with configural-relational short-term memory performance. J. Neurosci. 31, 7038–7042.

Quraan, M.A., Moses, S.N., Hung, Y., Mills, T., Taylor, M.J., 2011. Detection and localization of hippocampal activity using beamformers with MEG: A detailed investigation using simulations and empirical data. Human Brain Mapping 32, 812–827.

Styliadis, C., Ioannides, A.A., Bamidis, P.D., Papadelis, C., 2013. Amygdala responses to valence and its interaction by arousal revealed by MEG. Int J Psychophysiol.

Tesche, C.D., Karhu, J., 2000a. Theta oscillations index human hippocampal activation during a working memory task. Proc. Natl. Acad. Sci. U.S.A. 97, 919–924.

Tesche, C.D., Karhu, J.J., 2000b. Anticipatory cerebellar responses during somatosensory omission in man. Hum Brain Mapp 9, 119–142.
